# Supplementary material for: Hepatectomy versus Chemotherapy for Resectable Colorectal Liver Metastases in Progression after Perioperative Chemotherapy: Expanding the Boundaries of the Curative Intent
Source: Cancers (Basel). 2023 Jan 27;15(3):783. doi: 10.3390/cancers15030783 (PMC9913571; doi:10.3390/cancers15030783)
Supplement: Supplementary file 1 [file cancers-15-00783-s001.zip › cancers-2106633-supplementary.pdf]

SUPPLEMENTARY MATERIALS.

Supplementary table 1. Surgical data about patients submitted to hepatectomy after progression-disease.

	**HEP**
	27
**Timing of surgery (%)**	
**Synchronous liver and colon**	2 (7.4)
**Liver first**	3 (11.1)
**Colon first**	20 (74.1)
**N. cut surfaces (median [IQR])**	2.00 [1.00, 2.00]
**Clavien-Dindo (%)**	
**0**	16 (59.3)
**2**	7 (25.9)
**3**	2 (7.4)
**4**	1 (3.7)
